# Upcycling Scented Pandan Leaf Waste into High-Value Cellulose Nanocrystals via Ultrasound-Assisted Extraction for Edible Film Reinforcement

**DOI:** 10.3390/foods14091528

**Published:** 2025-04-27

**Authors:** Benjamard Rattanamato, Nattapong Kanha, Prem Thongchai, Kanyasiri Rakariyatham, Wannaporn Klangpetch, Sukhuntha Osiriphun, Thunnop Laokuldilok

**Affiliations:** 1 Faculty of Agro-Industry, Chiang Mai University, Chiang Mai 50100, Thailand; benjamard_rat@cmu.ac.th (B.R.);; 2 Office of Research Administration, Chiang Mai University, Chiang Mai 50200, Thailand

**Keywords:** scented pandan leaves, ultrasonic-assisted extraction, cellulose nanocrystals, response surface methodology, sweet potato starch, starch/CNC film, reinforcing agent

## Abstract

This study aims to optimize the parameters for the ultrasound-assisted extraction of cellulose nanocrystals (CNCs) from scented pandan leaf waste and to enhance the properties of edible films reinforced with CNC. The CNC extraction conditions were optimized using response surface methodology (central composite design) by varying two independent variables, including amplitude (25.86% to 54.14%) and ultrasonication time (11.89 min to 33.11 min). The optimal extraction conditions were 50% amplitude and 30 min ultrasonication, providing CNCs with the highest extraction yield (29.85%), the smallest crystallite size (5.85 nm), and the highest crystallinity index (59.32%). The extracted CNCs showed favorable physicochemical properties, including a zeta potential of −33.95 mV, an average particle diameter of 91.81 nm, and a polydispersity index of 0.26. Moreover, sweet potato starch (SPS)-based films incorporating various CNC concentrations (0, 2, 4, 6, and 8%) were fabricated. Increasing CNC concentrations improved key film properties, including thickness, moisture content, water vapor permeability, tensile strength, light transmittance, and color. Fourier transform infrared spectroscopy (FT-IR), X-ray diffraction (XRD), and scanning electron microscopy (SEM) analyses confirmed hydrogen bonding, crystallinity, and uniform CNC distribution within the film as CNC content increased. These findings highlight ultrasound-assisted extraction as an efficient method for producing high-quality CNCs from pandan leaf waste, offering sustainable nanofillers to enhance biodegradable edible films.

## 1. Introduction

Currently, the development of biobased materials to replace synthetic polymers is a topic that is receiving increasing attention. New materials from natural sources, especially non-wood fibers, which are a biodegradable and environmentally friendly source of cellulose, have been studied [1]. Cellulose nanocrystals (CNCs) are cellulose derivatives obtained through acid hydrolysis, which separates the crystalline and amorphous regions of cellulose fibers [2,3]. The crystalline is referred to as CNC, which possesses high mechanical strength, a large surface area, and enhanced crystallinity when dispersed in a suspension [4]. CNC has garnered attention as a reinforcing agent due to its nontoxicity, renewable nature, biodegradability, and mechanical and thermal properties [5,6]. The extraction of CNCs is commonly carried out using acid hydrolysis, where critical processing parameters, such as acid concentration, temperature, and reaction time, must be carefully controlled. Excessively high acid concentrations and prolonged reaction times can lead to the depolymerization of crystalline cellulose, resulting in a reduction in the yield and quality of the extracted CNCs [7]. Ultrasonic technology has been integrated into the extraction process to reduce these impacts and improve extraction efficiency. Using ultrasonic technology is an alternative method to enhance the yield and quality of CNCs via the cavitation phenomenon, which degrades polysaccharide linkages. The shock waves generated by ultrasonication enhance solvent diffusion, promoting the isolation and separation of micro- and nano-scale fibrils from cellulose sources [8,9] while reducing the environmental impact.

Recent studies have indicated agro-industrial biowastes as good raw materials for CNC extraction, including cucumber peel [10], banana peel [11], garlic skin and stalks [12,13], grape pomace [14], onion skin [15], pineapple peel [16], and sugarcane bagasse [17]. One particularly underutilized source of CNC is scented pandan (*Pandanus amaryllifolius* Roxb.) leaves, which are grown in Sri Lanka, Thailand, India, Malaysia, Indonesia, the Philippines, and probably in many other tropical and subtropical countries [18]. A scented pandan plantation can yield approximately 60 kg of fresh leaves per harvest or 6 tons per hectare annually [18]. The leaf extracts are rich in 2-acetyl-1-pyrroline (2AP), which is used as a flavoring agent in foodstuffs of many Asian countries [19]. However, about 76% of the residual pandan leaf fibers [20] remain unutilized after flavor extraction, contributing to agro-industrial biowaste and posing environmental challenges. Previous research by [8] reported that scented pandan fiber contains 48.8% cellulose, making it suitable for CNC extraction due to its renewability, biodegradability, and low cost. Despite this, no studies have been conducted on the extraction and application of CNCs from scented pandan leaves.

High-quality biopolymers, including polysaccharides, plant fibers, and proteins, are widely used in edible film production. Among them, starch is particularly attractive due to its excellent gel-forming and rheological properties, nontoxicity, low cost, and biodegradability [21]. Generally, starch-based films often exhibit poor water vapor barrier properties and low mechanical strength [22]. Zhang et al. [23] demonstrated that sweet potato starch had better film properties than other starches typically used for film preparation, including cassava and corn starches, and was incredibly suitable for making an edible film due to its high mechanical and water barrier properties. Interestingly, the incorporation of lignocellulosic materials, such as microcrystalline cellulose (MCC), has been shown to reinforce starch-based films, enhancing their mechanical and hydrophobic properties [24,25,26,27,28,29]. For instance, cassava starch films containing MCC or nanocellulose at concentrations of 0.5% and 2% showed improved mechanical strength and hydrophobicity [24]. Previously, CNCs from renewable sources, such as eucalyptus, sugarcane bagasse, and mango seed shell, have been used in starch films, and it was found that CNCs can improve the films’ water-vapor permeability and mechanical properties [30,31,32]. The integration of CNCs into sweet potato starch films offers a promising strategy to overcome these limitations, thereby expanding their potential applications within the food industry.

Among the advanced extraction technologies, ultrasound-assisted extraction (UAE) is a promising method for obtaining CNCs. This technique offers significant advantages in terms of speed, efficiency, energy consumption, and environmental sustainability [33]. Additionally, UAE can be effectively combined with various solvents, including water and acidified water [34]. It is essential to further clarify the conditions for extracting CNCs, particularly regarding the two primary parameters of UAE, namely amplitude and ultrasonication time. Therefore, this study aimed to optimize the UAE conditions for CNCs from scented pandan leaves using response surface methodology and to evaluate the properties of edible films reinforced with the extracted CNCs. The edible films were formulated using sweet potato starch (SPS) and varying CNC concentrations (0, 2, 4, 6, and 8%). Additionally, the physicochemical properties of both CNCs and the resulting edible films were comprehensively analyzed. The use of extracted CNCs in edible films might be able to improve film properties and is expected to serve as a reinforcing agent in various applications in the future.

## 2. Materials and Methods

### 2.1. Raw Materials and Chemicals

Scented pandan (*Pandanus amaryllifolius* Roxb.) leaves were cultivated in Uttaradit Province, Thailand. Leaves approximately 30 cm in length were selected and harvested during the rainy season (July 2020). The harvested leaves were thoroughly washed with clean water, cut into small pieces, and sun-dried for two days. The dried leaves were then packed in aluminum bags and stored in a glass desiccator at room temperature (~30 °C) until further use. The lignin, hemicellulose, and cellulose contents of the dried pandan leaves were determined following the method of [35].

Food-grade SPS was procured from PTK Solution and Supplies Co., Ltd., Bangkok, Thailand. All chemicals, including sodium hydroxide (NaOH), hydrogen peroxide (H_2_O_2_), sulfuric acid (H_2_SO_4_, 98% purity), and distilled water, were of analytical grade and were obtained from RCI LabScan, Bangkok, Thailand.

### 2.2. Extraction of Cellulose from Dried Scented Pandan Leaves

Cellulose was extracted from dried pandan leaves following the method of [10] with modifications. Cellulose pulp was isolated by soaking 20 g of dried pandan leaves in 500 mL of 20% (*w*/*v*) NaOH solution and heating with continuous stirring at 60 °C for 3 h using a magnetic stirrer (C-MAG HS 7, IKA Works (Thailand) Co., Ltd., Bangkok, Thailand). The solution was then neutralized by rinsing the pulp thoroughly with distilled water until the pH reached 7. Subsequently, 20 g of cellulose pulp was bleached using 15% (*v*/*v*) H_2_O_2_ solution (500 mL) under continuous stirring at 80 °C for 1 h using a magnetic stirrer (IKA Works (Thailand) Co., Ltd.). The bleaching step was repeated three times to ensure maximum removal of impurities. The bleached cellulose pulp was then washed several times with distilled water until a neutral pH was achieved. Finally, the cellulose pulp was dried at 60 °C for 24 h, packed in an aluminum bag, and stored in a glass desiccator at room temperature (~30 °C) until further use.

### 2.3. Optimization of UAE Conditions for CNCs

#### 2.3.1. Experimental Design, Mathematical Modeling, Fitting, and Statistical Analysis

UAE was employed to extract CNCs from scented pandan leaves. A central composite design (CCD) was used to optimize the UAE conditions. Two independent variables, namely amplitude (X_1_) and ultrasonication time (X_2_), were investigated in relation to three dependent variables, including yield, crystallite size, and crystallinity index. The values of X_1_ and X_2_ ranged from 25.86% to 54.14% and from 11.89 min to 33.11 min, respectively. The experimental design included two axial points and four center points, resulting in a total of twelve experimental combinations, as summarized in Table 1. The experimental runs were randomized to minimize variability due to uncontrollable factors [36]. The relationship between the dependent (Y) and independent (X) variables was modeled using the following quadratic polynomial equation (Equation (1)):

Y = *β*_0_ + *β*_1_X_1_ + *β*_2_X_2_ + *β*_3_X_1_^2^ + *β*_4_X_2_^2^ + *β*_5_X_1_X_2_ (1)

where *β*_0_ is the constant coefficient, *β*_1_ and *β*_2_ are the linear regression coefficients, *β*_3_ and *β*_4_ are the quadratic regression coefficients, and *β*_5_ is the regression coefficient for the interaction effects. Design-Expert software version 13.0.5.0 (Stat-Ease Inc., Minneapolis, MN, USA) was used to determine the coefficient of determination (R^2^), assess model fit, optimize conditions using the desirability function, and generate three-dimensional response surface plots. The predicted values were calculated using the polynomial equation above. A model was deemed significant if the *p*-value was below 0.05, and the lack of fit was considered non-significant if the *p*-value exceeded 0.05, indicating an adequate model fit [36].

#### 2.3.2. UAE of CNC from Scented Pandan Leaves

The CNC extraction process followed the method of [37] with modifications. Here, 10 g of bleached cellulose pulp was digested in 250 mL of 50% (*v*/*v*) sulfuric acid while maintaining the reaction mixture in an ice bath for 3 h. The reaction was quenched by adding 2000 mL of cold distilled water, and the mixture was allowed to precipitate overnight at 4 °C. The precipitate was subsequently washed with distilled water to remove residual acids before being centrifuged using a Himac centrifuge (CR22N, Eppendorf Himac Technologies Co., Ltd., Ibaraki, Japan) at 8000 rpm for 15 min. This centrifugation process was repeated until the sample suspension appeared milky white. The sample was then subjected to ultrasonic treatment using a probe ultrasonicator (VCX500, Sonics & Materials, Inc., Newtown, CT, USA) operating at 500 W and a 20 kHz frequency. The amplitude and ultrasonication time were set as specified in Section 2.3.1. Following ultrasonication, the suspension was centrifuged again at 8000 rpm for 15 min before being freeze-dried (Freezezone 4.5, Labconco Corporation, Kansas City, MO, USA) at −40 °C for 48 h, yielding CNC powder. The CNC powder was then packed in aluminum bags and stored at −18 °C until further analysis.

The optimal UAE conditions were determined to maximize yield, minimize crystallite size, and achieve the highest crystallinity index. Once the optimal conditions were identified, CNC powders were prepared accordingly, and the actual experimental values were compared with the predicted values.

#### 2.3.3. Analysis of CNC Properties

##### Yield of CNCs

The yield of CNCs was determined according to the method of [38] by calculating the ratio between the final mass of dried CNC powder (W_2_) and the initial mass of dried pandan leaves (W_1_), using the following equation (Equation (2)):

Yield (%) = (W_2_/W_1_) × 100 (2)

##### Crystallinity Index and Crystallite Size

The crystallinity index and crystallite size were analyzed using an X-ray diffractometer (MiniFlex 600, Rigaku Holdings Corporation, Tokyo, Japan) with Cu-Kα radiation (λ = 0.154 nm) at a voltage of 40 kV and a current of 30 mA [31]. CNC diffraction patterns were recorded over a scanning range of 2θ = 10–60°, with a step size of 0.02° and a scan speed of 0.02°/s. The crystallinity index was calculated using the following Segal equation [39] (Equation (3)):

Crystallinity index = [(*I*_002_ − *I*_am_)/(*I*_002_)] × 100 (3)

where *I*_002_ is the highest intensity of the crystalline peak at 2θ = 22°; *I*_am_ is the intensity 2θ = 16°, denoting the amorphous region’s diffraction intensity.

The average crystallite size was calculated using the following Scherrer equation [40] (Equation (4)):

D = 0.89λ/*β*cosθ_B_ (4)

where D is the size of the crystal (nm). *λ* is the wavelength of the X-ray source (Cu-K_α_ = 0.15406 nm). *β* is the full width at the half-maximum height of the primary diffraction peak in radians. θ_B_ is the Bragg angle in radians equal to half of 2θ.

##### Fourier Transform Infrared (FT-IR) Spectroscopy

The FT-IR spectra of scented pandan leaves, unbleached cellulose, bleached cellulose, and CNCs were analyzed using the potassium bromide (KBr) disc method. Finely ground solid samples (2 g) were mixed with KBr powder and compressed into a disc using a hydraulic press. Infrared spectra were recorded using an FT-IR spectrometer (FTIR-4700, JASCO, Hertfordshire, UK) over a wavenumber range of 4000–400 cm^−1^, with a resolution of 4 cm^−1^, and expressed in transmittance mode.

##### X-Ray Diffraction (XRD)

The XRD analysis followed the operating conditions described by [31], with some modifications. The XRD patterns were obtained using an X-ray diffractometer (Rigaku Holdings Corporation), with Cu-K_α_ radiation (λ = 1.549 Å) at an operating voltage of 40 kV and a current of 30 mA. The diffraction patterns were recorded over a 2θ range of 10–60° with a scanning speed of 0.02°/s.

##### Zeta Potential and Polydispersity Index

The CNC sample was prepared as suspensions at a concentration of 0.5% (*w*/*v*), and their zeta potential and polydispersity index were measured using a Zetasizer (ZSU5800, Malvern Instruments Ltd., Worcestershire, UK) at 25 °C in fully automated mode.

### 2.4. Preparation of SPS Film Reinforced with CNC

SPS and CNCs extracted from the optimized conditions were used for film preparations, followed by the method of [41] with modifications. A 4% (*w*/*v*) SPS solution was prepared in distilled water and homogenized with a magnetic stirrer (IKA Works (Thailand) Co., Ltd.) for 10 min at room temperature. CNCs, at concentrations of 0, 2, 4, 6, and 8% (*w*/*w* starch), were then incorporated into the solution and mixed for 15 min until homogeneous. The mixture was subsequently heated at 100 °C for 30 min, resulting in a transparent and viscous solution. Glycerol (20% of the total solids) was added and stirred at room temperature until a homogeneous mixture was obtained. The solution (20 g) was cast into a 140 × 140 mm Petri dish and dried in a hot air oven (UF110, Memmert GmbH + Co.KG, Schwabach, Germany) at 40 °C until a thin film formed. The film was carefully peeled from the dish, packed in an aluminum bag, and stored in a desiccator at room temperature with 52% relative humidity for at least 16 h before further analysis.

### 2.5. Characterization of Films

#### 2.5.1. Characterization of Film Structural Properties 

##### FT-IR Analysis of Films

FT-IR analysis was performed following [30]. Film samples were prepared using the KBr disc method. Their FT-IR spectra were recorded over a wavenumber range of 4000–400 cm^−1^ with a resolution of 4 cm^−1^ using an FT-IR spectrometer (JASCO) in transmittance mode.

##### XRD Analysis of Films

XRD patterns of the films were analyzed using an X-ray diffractometer (Rigaku Holdings Corporation) with Cu-K_α_ radiation (λ = 1.549 Å) at 40 kV and 30 mA [31]. Scanning was performed over a 2θ range of 10–90° with a step size of 0.02°/s.

##### Scanning Electron Microscopy (SEM)

The surface morphology of film samples (0.4 mm × 0.4 mm) was examined using a scanning electron microscope (Prisma E, Thermo Fisher Scientific Inc., Waltham, MA, USA), operated under vacuum with an accelerating voltage of 15 kV.

#### 2.5.2. Physical and Chemical Properties

##### Film Thickness

Film thickness was measured following the method described by [42] with modifications. Film samples (9 cm × 9 cm) were cut, and thickness was measured at the center and four corners in triplicate.

##### Moisture Content

Moisture content was determined following the method of [38]. Film samples were dried in a UF110 air-circulation oven (Memmert GmbH + Co.KG) at 105 °C. The moisture content was calculated based on the mass difference before and after drying.

##### Water Vapor Permeability (WVP)

The WVP of the film samples was determined according to ASTM E96-95 standards [43]. Film samples (7.5 cm × 7.5 cm) were mounted on WVP cups (2.5 cm depth, 6.8 cm diameter) containing 18 mL of distilled water. The cups were sealed and placed in a humidity chamber (FX 1077, Jeio Tech Co., Ltd., Ansan, Republic of Korea) at 25 °C and 50% relative humidity. Weight changes were recorded hourly for 8 h. The WVP was calculated using the following Equation (5):

WVP (g·m/m^2^·Pa·s) = (∆*W* × *L*)/(*t* × *A* × Δ*P*) (5)

where *W* is the weight increase of the test vessel (g), *L* is the film thickness (m), *t* is the time (s) for weight increase, *A* is the film permeation area (m^2^), and Δ*P* is 2339 Pa at 20 °C.

##### Mechanical Properties Analysis

Tensile strength (TS) and elongation at break (EB) of the film samples were determined following ASTM Standard D 882-02 [44] using a universal testing machine (H1K-S, Tinius Olsen TMC, Horsham, PA, USA). Film samples (10 mm × 15 mm) were clamped with an initial grip separation of 50 mm and subjected to a tensile speed of 5 mm/min. TS and EB were calculated using the following Equations (6) and (7):

TS (MPa) = *F*/(*Χ* × *W*) (6)

EB (%) = (∆*L*/*L*_0_) × 100 (7)

where *F* is the stress of the film at break (N), *W* is the film width (mm), Δ*L* is the elongated length (mm), and *L*_0_ is the original length (mm) of the film sample.

##### Light Transmittance Analysis

The light transmittance of the film samples was analyzed using a UV–Vis spectrophotometer (Dynamica HALO XB-10, Dynamica Scientific Ltd., Hong Kong, China) following the method described by [45]. The transmittance of film samples (50 × 30 mm) was measured over the wavelength range of 200–800 nm. A blank measurement was conducted using an empty test cell.

##### Color

Film color parameters (L*, a*, b*) were measured using a Konica Minolta colorimeter (CR-400, Konica Minolta, Inc., Tokyo, Japan). Chroma (C*) and hue angle (*h°*) were derived from a* and b* values. The total color difference (ΔE*) and whiteness index (WI) were calculated using the following Equations (8) and (9) [46], based on the standard white plate parameters (L* = 98.09, a* = 0.40, b* = 1.02):

ΔE* = [(ΔL*)^2^ + (Δa*)^2^ + (Δb*)^2^]^0.5^ (8)

WI = 100 − [(100 − L*) + (a*)^2^ + (b*)^2^]^0.5^ (9)

##### Statistical Analysis

All experiments were carried out in triplicate. One-way analysis of variance (ANOVA) was conducted, and significant differences among means were determined using Duncan’s multiple range test. Statistical analysis was performed using the Statistical Package for the Social Sciences (SPSS, Version 17, SPSS Inc., Chicago, IL, USA) at a significance level of *p* ≤ 0.05.

## 3. Results and Discussion

### 3.1. Model Fitting and Response Surface Analysis

The yield (23.45–26.98%), crystallite size (6.35–13.48 nm), and crystallinity index (35.75–62.86%) of CNCs obtained from the UAE of scented pandan leaves are presented in Table 1. The quadratic models (Equations (10)–(12)) for amplitude and ultrasonication time were statistically significant (*p* ≤ 0.05) for all responses, with relatively high regression coefficient (R^2^) values for yield (0.8477), crystallite size (0.9030), and crystallinity index (0.9970) for Equations (10)–(12), respectively. These findings indicate a strong correlation between UAE parameters and the predicted responses.

Yield = 36.27 + 24.04X_1_ + 4.43X_2_ + 5.76X_1_X_2_ + 2.03X_1_^2^ + 2.50 × 10^2^X_2_^2^ (10)

Crystallite size = 57.41 − 47.45X_1_ − 7.84X_2_ + 2.25 × 10^2^X_1_X_2_ + 0.84X_1_^2^ − 0.84X_2_^2^ (11)

Crystallinity index = 421.85 + 365.35X_1_ + 9.02X_2_ + 40.96X_1_X_2_ − 0.44X_1_^2^ + 5.21X_2_^2^ (12)

**Figure 1:**
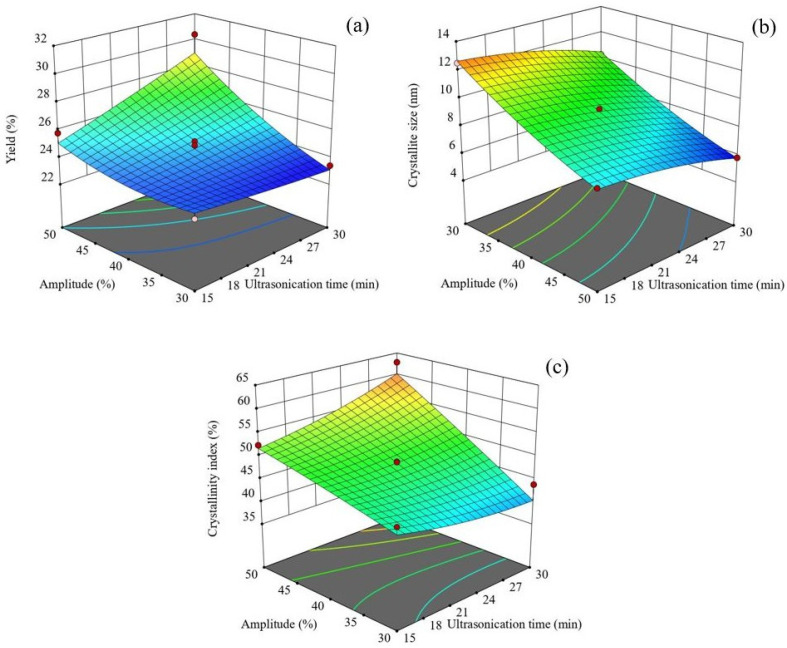
Response surface plots of ultrasonic amplitude (%) and extraction time (min) on yield (**a**), crystallite size (**b**), and crystallinity index (**c**).

The effect of amplitude and ultrasonication time on CNC yield is shown in Figure 1a. Higher amplitude levels resulted in increased CNC yield due to intensified cavitation, which generates microjets, shock waves, and high shear forces [47,48,49,50]. Cavitation disrupts glycosidic bonds within the cellulose polymer, facilitating CNC release [51]. However, at an amplitude of 30%, prolonged ultrasonication slightly reduced CNC yield, probably due to particle agglomeration [49]. Excessive ultrasonication at lower amplitudes may lead to aggregation, as the increased specific surface area and hydrogen bonding between crystallites promote clustering [52]. Moreover, sediment formation during centrifugation may contribute to yield loss. In contrast, extending ultrasonication time at higher amplitudes significantly increased CNC yield, suggesting that the increased cavitation energy mitigates aggregation effects. At high amplitudes, CNC solubility in water improved due to enhanced liquid penetration and diffusion within the cellulose matrix, facilitating bond breakage and CNC regeneration.

Figure 1b shows the influence of amplitude and ultrasonication time on CNC crystallite size. Increasing both parameters led to a reduction in crystallite size, attributed to glycosidic bond cleavage in cellulose chains. In [50], the authors demonstrated that higher amplitudes and prolonged ultrasonication significantly shorten CNC length due to cavitation [51]. Consequently, the application of the maximum amplitude and ultrasonication time resulted in the greatest reduction in crystallite size.

Interestingly, while increasing the ultrasonication amplitude reduced the crystallite size of CNCs, it had a different effect on the crystallinity index, as shown in Figure 1c. The results indicated that higher amplitudes led to an increase in the crystallinity index of CNCs. This finding is consistent with the observations of [49], who reported that the crystallinity of nanocellulose increases with increasing vibration amplitude. The enhanced structural disintegration at higher amplitudes likely facilitates the release of more CNC crystallites, as longer cellulose chains are broken into smaller segments in both amorphous and crystalline regions due to the implosive collapse associated with high-intensity sonication. Conversely, when ultrasonication was performed at approximately 30% amplitude, the crystallinity index of CNC decreased with increasing sonication time. This reduction can be attributed to the aggregation of individual CNC particles, driven by intermolecular hydrogen bonding. Similar findings were reported by [49], who suggested that the rupture of hydrogen bonds leads to the collapse of the crystalline structure of nanocellulose, thereby reducing its crystallinity. However, as ultrasonication time increased at higher amplitudes, the breakdown of amorphous regions in cellulose became more pronounced, facilitating the release of additional CNC crystallites and resulting in a higher crystallinity index. Contrastingly, ref. [50] observed that excessive ultrasound energy could also damage crystalline regions, leading to a decrease in the CNC crystallinity index. In this study, the interaction between amplitude and ultrasonication time played a role in increasing the crystallinity index of CNCs. This observation aligns with the CNC yield data (Figure 1a), where the highest crystallinity index at maximum amplitude and sonication time was attributed to the increased release of CNC crystallites.

### 3.2. Optimization of UAE Conditions and Model Validation

The optimal conditions for providing the highest CNC yield, smallest crystallite size, and maximum crystallinity index resulted in a desirability score of 0.89. These conditions were 50% amplitude and a 30 min ultrasonication time. The predicted responses under these conditions were a yield of 28.95%, a crystallite size of 5.78 nm, and a crystallinity index of 60.19%. To validate these predictions, an experimental run was conducted under the optimized conditions. The experimental results obtained were a yield of 29.85%, a crystallite size of 5.85 nm, and a crystallinity index of 59.32%. The close agreement between the experimental and predicted values indicated minimal variability, with the experimental yield and crystallite size exceeding the predicted values by 3.02% and 1.20%, respectively, while the experimental crystallinity index was 1.45% lower than the predicted value. The CNC powders produced under these optimized conditions were subsequently characterized in terms of their physicochemical properties compared with their raw material, unbleached cellulose, and bleached cellulose, and used in the fabrication of edible films from sweet potato starch.

### 3.3. CNC Properties

#### 3.3.1. Cellulosic and Non-Cellulosic Components in Scented Pandan CNCs

Table 2 shows the cellulosic and non-cellulosic components of CNCs extracted by UAE. The CNCs consisted of 83.79% cellulose, with negligible hemicellulose and lignin, indicating high purity. Minimal residual chemical components, such as moisture, carbohydrates, proteins, and ash were present, but their low concentrations did not influence the CNC properties.

#### 3.3.2. Zeta Potential

The CNCs exhibited negative surface charges, with a zeta potential of −33.95 mV, comparable to the values reported for CNCs derived from date palm stem waste (−29.3 mV) and tea stalks (−33.4 mV) [53,54]. Zeta potential values exceeding ± 30 mV indicate sufficient electrostatic repulsion to maintain suspension stability [54,55]. Hence, a zeta potential below −30 mV suggests that CNC aggregation or flocculation is unlikely [55].

#### 3.3.3. Average Particle Diameter and Polydispersity Index

Dynamic light scattering (DLS) analysis revealed an average CNC particle diameter of 91.81 nm, within the range reported for CNCs from date palm stem waste (45.00–78.40 nm) and sawdust waste (101.36–107.48 nm) [54,56]. The extracted CNCs exhibited a polydispersity index (PDI) of 0.26, similar to the PDI of CNCs derived from date palm stem waste (0.27). A lower PDI suggests a narrow particle size distribution, indicative of a monodisperse system [57]. However, for polymer-based nanomaterials, a PDI below 0.2 is generally preferred [58].

#### 3.3.4. FT-IR Analysis

**Figure 2:**
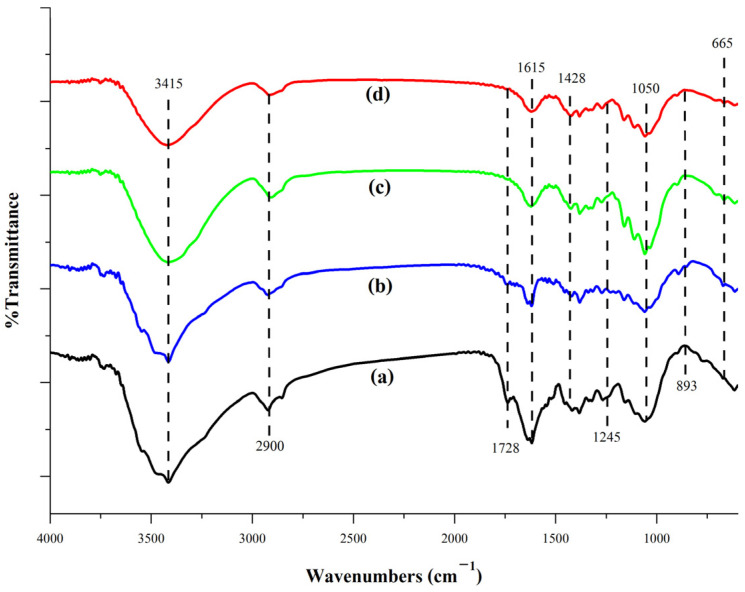
FT-IR spectra of scented pandan leaves (**a**), unbleached cellulose (**b**), bleached cellulose (**c**), and CNCs (**d**).

The FT-IR spectra of scented pandan leaves, unbleached cellulose, bleached cellulose, and CNCs are shown in Figure 2. The FT-IR analysis was conducted to evaluate changes in functional groups and their interactions throughout the CNC extraction process. All samples exhibited characteristic cellulose peaks at 665, 893, and 1050 cm^−1^ [54]. The peak at 2900 cm^−1^ corresponds to the C–H stretching vibrations in the glucose units of cellulose. Bleached cellulose and CNCs lack a sharp peak at 3415 cm^−1^ (associated with N–H stretching of amide A) and a small peak at 1637 cm^−1^ (indicative of C=O stretching vibrations of amide I) [59]. This absence suggests that proteins were effectively removed during the bleaching process, leaving only a broad peak in the 3500–3200 cm^−1^ range, which represents the –OH stretching vibrations of cellulose and intermolecular hydrogen bonding [35]. Furthermore, the absence of an absorption band at 1728 cm^−1^ in bleached cellulose and CNCs indicates the successful removal of carbonyl groups and ester linkages associated with carboxylic groups in lignin and hemicellulose during bleaching [54]. These findings confirm that non-cellulosic components were effectively eliminated while preserving the cellulose structure during CNC extraction.

#### 3.3.5. XRD Analysis

**Figure 3:**
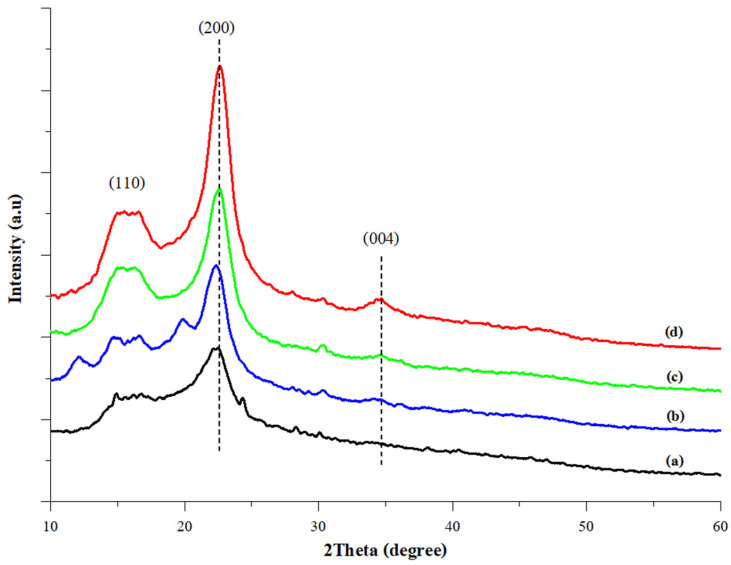
The XRD spectra of scented pandan leaves (**a**), unbleached cellulose (**b**), bleached cellulose (**c**), and CNCs (**d**).

The XRD patterns of scented pandan leaves, unbleached cellulose, bleached cellulose, and CNCs are presented in Figure 3. All samples exhibited diffraction peaks at approximately 2θ = 16.6°, 22.5°, and 34.7°, corresponding to the 110, 200, and 004 crystal planes of cellulose, respectively [60,61]. This confirms that the crystalline structure of cellulose was maintained throughout the UAE-based CNC extraction process. The intensity of the diffraction peaks progressively increased at each stage of CNC extraction. This enhancement is attributed to the removal of non-cellulosic components and the increased cleavage of glycosidic bonds in cellulose fibers during UAE, which contributed to the higher purity of CNC’s crystalline structure.

### 3.4. Influences of CNC Incorporation on Sweet Potato Starch-Based Film Properties

#### 3.4.1. Structural Properties of Films

**Figure 4:**
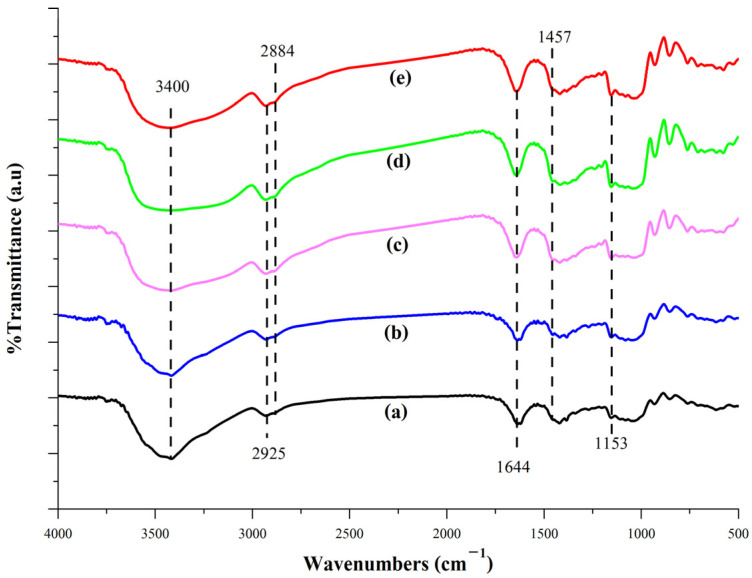
FT-IR spectra of sweet potato starch (SPS)-based film (**a**) and its blend film containing CNCs at concentrations of 2% (SPS + 2% CNC film; (**b**)), 4% (SPS + 2% CNC film; (**c**)), 6% (SPS + 2% CNC film; (**d**)), and 8% (SPS + 2% CNC film; (**e**)).

**Figure 5:**
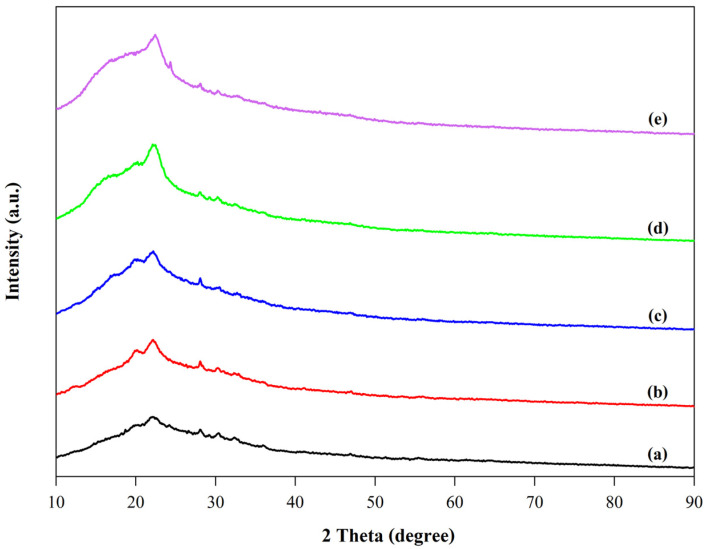
XRD spectra of sweet potato starch (SPS)-based film (**a**) and its its blend film containing CNCs at concentrations of 2% (SPS + 2% CNC film; (**b**)), 4% (SPS + 2% CNC film; (**c**)), 6% (SPS + 2% CNC film; (**d**)), and 8% (SPS + 2% CNC film; (**e**)).

**Figure 6:**
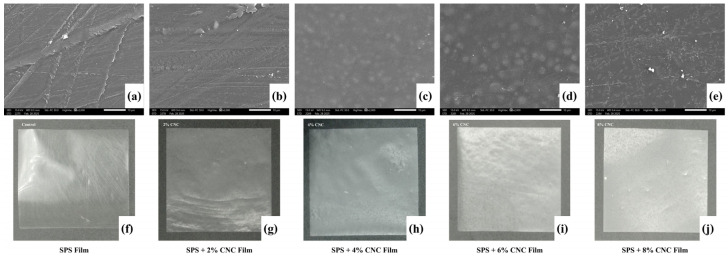
SEM images and appearances of sweet potato starch (SPS)-based film (**a**,**f**) and its its blend film containing CNCs at concentrations of 2% (SPS + 2% CNC film; (**b**,**g**)), 4% (SPS + 2% CNC film; (**c**,**h**)), 6% (SPS + 2% CNC film; (**d**,**i**)), and 8% (SPS + 2% CNC film; (**e**,**j**)).

The structural properties of sweet potato starch (SPS)-based films reinforced with varying concentrations (0, 2, 4, 6, and 8%) of scented pandan CNCs are shown in Figure 4, Figure 5 and Figure 6. These films are designated as SPS film, SPS + 2% CNC film, SPS + 4% CNC film, SPS + 6% CNC film, and SPS + 8% CNC film, respectively.

##### FT-IR

The FT-IR spectra of the film samples within the wavenumber range of 4000–500 cm^−1^ reveal notable similarities due to their common SPS-based composition (Figure 4).

The spectra show a peak at 3400 cm^−1^, corresponding to the O–H stretching vibrations present in all samples. The intensity of this peak increases in the blend films containing 4–8% CNCs compared to the blend film with 2% CNCs and the SPS film, suggesting a higher concentration of O–H groups due to CNC incorporation. Moreover, the O–H stretching vibration appears as a broad band that shifts in both directions, a blue shift from 3400 to 3600 cm^−1^, indicative of higher vibrational frequencies, and a red shift from 3400 to 3100 cm^−1^, representing lower vibrational frequencies in the blend films with 4–8% CNC. These shifts suggest a significant increase in both inter- and intramolecular hydrogen bonding within the CNC-incorporated films. In addition, peaks around 2900 cm^−1^ are attributed to C–H stretching vibrations associated with both starch and cellulose [62]. The peak at 1640 cm^−1^ corresponds to the bending vibrations of –OH groups [63] and is influenced by water absorption within the starch-based film. Lastly, characteristic peaks associated with glycosidic bonds in starch and cellulose are observed at approximately 800–950 cm^−1^ and 1150 cm^−1^, indicating the presence of α- and β-configurations of glycosidic linkages, respectively [62,64]. The incorporation of CNC resulted in a slight increase in the intensity of peaks related to C–H stretching vibrations, O–H bending vibrations, and glycosidic bonds.

##### XRD

The XRD patterns of the film samples are shown in Figure 5. The SPS film showed three prominent diffraction peaks, a shoulder peak at approximately 2θ = 19°, a strong peak at 2θ = 22°, and a smaller peak at 2θ = 24°. This diffraction pattern is characteristic of CA-type starch [65]. The incorporation of CNCs into the SPS film led to an increment of two diffraction peaks, namely the shoulder peak at 2θ = 15° and the strong peak at 2θ = 22°, which correspond to the characteristic peaks of CNCs (Figure 3). The intensity of both peaks increased with higher CNC concentrations, indicating an improvement in the film’s crystallinity due to CNC reinforcement. Similar results were also observed in CNC-containing films prepared with chitosan or carboxymethyl cellulose [66,67]. The incorporation of CNCs’ rigid molecules into the film matrix facilitated the orientation of the polymeric molecules and enhanced the formation of hydrogen bonds between the polymer chains [67]. These interactions can impact the arrangement of crystalline regions and influence diffraction behavior.

##### Surface Morphology

The SEM images and appearances of the film samples are presented in Figure 6a–e. The surface morphology of the SPS film and the film containing 2% CNCs exhibited a smooth surface without visible particles, as shown in Figure 6a,b. The linear patterns observed on the film surface resulted from the contact surface of the container used during film preparation. In contrast, films incorporating 4% to 8% CNCs displayed distinct surface morphologies characterized by white circular features dispersed across the entire film surface, as observed in Figure 6c–e. The film with 6% CNCs exhibited larger white circles compared to those in the films with 4% and 8% CNCs. These white circles are likely CNC aggregates distributed throughout the film. The smaller aggregates were observed in the film containing 8% CNCs. Higher CNC concentrations could increase the likelihood of aggregation as particles have more interaction opportunities. A similar result was also found in gelatin film containing CNCs [68]. However, the SEM image of the film containing 8% CNCs might directly reveal the aggregation behavior of CNCs, which could form strong interactions through hydrogen bonds or electrostatic forces, becoming a compact structure of aggregated CNCs as a short rod-like structure dispersed on the film surface. This behavior might be associated with the surface charge of CNCs [69,70]. Furthermore, the aggregated CNCs could result in light scattering and reduced transparency. Figure 6f–j presents photographs of the film’s appearance. Overall, all film samples showed a similar physical appearance, with a glossy and reflective surface. The SPS film had relatively high transparency, a characteristic typical of starch-based films. However, with increasing CNC content, the films became more opaque and whiter. CNCs appeared to be evenly dispersed across all blend films.

#### 3.4.2. Physical and Chemical Properties of the Films

##### Film Thickness and Moisture Content

The film thickness and moisture content of the films reinforced with CNCs are summarized in Table 3. Film thickness is one of the key parameters influencing mechanical properties and water vapor permeability [71,72,73]. The thickness of the films ranged from 48.56 to 52.60 µm. A significant increase in thickness (*p* ≤ 0.05) was observed with higher CNC content, consistent with previous studies attributing this trend to the increased solid content in the film matrix [73,74]. Moreover, the negative surface charge of CNCs might enhance repulsive forces between CNC chains, leading to structural expansion and improved drying efficiency. An inverse relationship was observed between film thickness and moisture content, wherein increasing CNC content resulted in reduced moisture content. This suggests that CNC incorporation enhances intermolecular repulsion, promoting film swelling and facilitating moisture transfer during the hot air drying process. Consequently, a significant reduction in moisture content (*p* ≤ 0.05) was noted as the CNC concentration increased. The moisture content of the films in this study ranged from 2.15% to 9.43%, which was relatively low compared to CNC-reinforced edible films reported in other studies (9.62% to 15.26%) [74,75].

##### Water Vapor Permeability

The effect of CNC concentration on the water vapor permeability (WVP) of the edible films is presented in Table 3. The WVP values significantly decreased with increasing CNC content (*p* ≤ 0.05), indicating improved barrier properties against water vapor transmission. Three mechanisms may explain how CNC incorporation improves the WVP of edible films, namely (1) the formation of a hydrogen-bonded network, (2) an increase in tortuosity, and (3) enhanced hydrophobicity [38,76,77]. First, CNCs can create hydrogen bonds with the polymer matrix, resulting in a more stable and less porous structure [76]. Second, the high crystallinity of CNCs might act as a hydrophobic barrier, forcing water vapor molecules to navigate a more tortuous path through the film matrix, thereby increasing the diffusion path length [74,78,79]. Lastly, CNCs could enhance the hydrophobicity of the film, rendering it less permeable to water [77]. In addition, the film reinforced with 8% CNCs exhibited the lowest WVP value (68.83 × 10^−3^ g·mm/m^2^·h·kPa) (*p* ≤ 0.05), probably due to its increased thickness and extended diffusion pathway.

##### Mechanical Properties

The mechanical properties of edible films reinforced with varying concentrations of CNCs are shown in Table 3. All CNC-incorporated films exhibited higher tensile strength than the SPS film (a film without CNCs). Increasing the CNC content significantly increased the tensile strength of the films (*p* ≤ 0.05), consistent with previous studies on CNCs derived from bacteria [74], areca nut pulp [80], and corn husks [41]. The high surface area of CNCs, enriched with free hydroxyl groups, facilitates strong intermolecular hydrogen bonding with the hydroxyl groups in the SPS matrix (composed of amylose and/or amylopectin). As the CNC content increased, the interactions intensified, contributing to improved tensile strength. On the other hand, elongation at break, an indicator of film flexibility, decreased significantly with increasing CNC content (*p* ≤ 0.05), indicating that higher CNC concentrations resulted in stiffer and less flexible films. This trend is consistent with findings from other CNC-incorporated films [41,74,80]. According to [64], the rigid network formed by nanocellulose and starch restricts the movement of starch molecular chains, thereby reducing elongation at break. These results demonstrated that CNCs effectively reinforced SPS film by enhancing its resistance to tensile forces while reducing flexibility.

##### Light Transmittance

Light transmittance is a crucial property influencing the application of edible films, as it determines the film’s ability to transmit light without scattering [81]. The SPS film exhibited the highest light transmittance (91.80%), indicating minimal light-blocking capability (Table 3). In contrast, CNC-incorporated SPS films showed lower light transmittance values, ranging from 67.97% to 85.21%, with the significant reduction observed as the CNC content increased (*p* ≤ 0.05). The results suggest that CNC incorporation enhances light-blocking properties. The reduction in light transmittance might be attributed to interactions between CNC and the SPS matrix, which promote increased crystallinity, leading to alterations in the refractive index and subsequently affecting light transmission [82]. Similar trends have been reported in CNC-reinforced films derived from areca nut pulp [80], corn husk [41], and mulberry pulp [83]. These findings highlight the potential application of CNC-reinforced films as edible packaging materials for light-sensitive food products.

##### Color Properties

Film color is a critical perceptual attribute influencing consumer acceptance. In this study, the color properties of SPS films reinforced with different CNC concentrations were evaluated based on lightness (L*), whiteness index (WI), and total color difference (ΔE*). Table 3 shows the color characteristics of all edible film samples. The SPS film exhibited the lowest L* value (*p* ≤ 0.05), whereas CNC incorporation significantly increased L* values in the films (*p* ≤ 0.05), resulting in a brighter appearance. The SPS film had the lowest WI value, and higher CNC concentrations led to significant increases in WI values (*p* ≤ 0.05). This suggests that CNC addition reduces UV-light absorption, producing a whiter appearance. The L* and WI values together confirmed the bright white coloration of the blend films, as depicted in Figure 6. Additionally, ΔE* was analyzed to quantify the color difference between the SPS film and each CNC-reinforced film. The ΔE* values significantly increased with CNC content (*p* ≤ 0.05), indicating that CNC incorporation influenced the film’s color. Similar trends have been reported in CNC-reinforced films derived from other sources [41,74].

## 4. Conclusions

This study successfully extracted CNCs from scented pandan leaves using UAE. The optimal conditions for maximizing CNC yield (29.85%), minimizing crystallite size (5.85 nm), and achieving the highest crystallinity index (59.32%) were determined to be 50% amplitude and an ultrasonication time of 30 min. Characterization of the extracted CNCs, including zeta potential, average particle diameter, and polydispersity index, indicated that the CNCs exhibited negative surface charges, an average particle diameter of 91.81 nm, and a particle size distribution characteristic of a monodisperse system. FT-IR and XRD analyses confirmed the effective removal of non-cellulosic components during the UAE process and validated the preserved crystalline structure of the CNCs. Furthermore, the incorporation of 8% CNCs into sweet potato starch (SPS) film (SPS + 8% CNC film) significantly enhanced its properties. This film exhibited superior barrier performance against water vapor, improved mechanical strength, better light blocking capabilities, and highly uniform CNC dispersion within the SPS matrix. FT-IR and XRD analyses further indicated that the increased CNC content improved film properties due to enhanced hydrogen bonding and crystallinity. These findings suggest that scented pandan CNCs hold substantial potential as a reinforcing agent in various applications, including edible films, food packaging, and nanocomposite materials.

## Figures and Tables

**Table 1:** Yield, crystallite size, and crystallinity index of CNC from scented pandan leaves.

Treatment	X_1_ (%)	X_2_ (min)	Yield (%)	Crystallite Size (nm)	Crystallinity Index (%)
1	30	15	23.6	12.50	46.09
2	50	15	25.8	7.72	52.36
3	30	30	23.4	10.26	43.79
4	50	30	30.4	5.83	62.86
5	25.8579	22.5	23.6	13.48	35.75
6	54.1421	22.5	26.9	6.35	56.06
7	40	11.8934	23.7	9.83	48.13
8	40	33.1066	24.8	7.12	48.34
9	40	22.5	24.4	9.00	48.41
10	40	22.5	25.2	9.09	48.21
11	40	22.5	24.9	9.28	48.83
12	40	22.5	24.1	9.09	48.68

**Table 2:** Cellulosic and non-cellulosic components in scented pandan CNCs.

Sample	Cellulose (%)	Hemicellulose (%)	Lignin (%)
Scented pandan leaves	44.45 ± 0.63 ^d^	19.94 ± 0.52 ^a^	13.67 ± 0.99 ^a^
Unbleached cellulose	54.07 ± 0.15 ^c^	12.67 ± 0.58 ^b^	12.21 ± 0.32 ^b^
Bleached cellulose	69.55 ± 0.17 ^b^	6.79 ± 0.13 ^c^	5.54 ± 0.53 ^c^
CNCs	83.79 ± 0.21 ^a^	-	-

Different superscript letters in each column indicate significant differences at *p* ≤ 0.05.

**Table 3:** Properties of edible films reinforced with varying concentrations of CNCs.

Edible Films	Thickness (mm)	Moisture (%)	WVP (×10^−3^ g·mm/m^2^·h·kPa)	Tensile Strength (MPa)	Elongation at Break (%)	%T	L*	WI	∆E*
SPS	0.486 ± 0.005 ^d^	9.43 ± 0.15 ^a^	79.16 ± 0.17 ^a^	0.38 ± 0.05 ^c^	42.65 ± 0.24 ^a^	91.80 ± 0.02 ^a^	89.90 ± 0.05 ^d^	89.86 ± 0.05 ^d^	0.05 ± 0.03 ^e^
SPS + 2% CNC	0.509 ± 0.002 ^c^	8.42 ± 0.21 ^b^	75.25 ± 0.31 ^b^	0.45 ± 0.02 ^c^	39.07 ± 0.90 ^b^	85.21 ± 0.03 ^b^	91.20 ± 0.09 ^c^	91.15 ± 0.09 ^c^	1.28 ± 0.09 ^c^
SPS + 4% CNC	0.517 ± 0.002 ^b^	5.69 ± 0.24 ^c^	71.48 ± 0.45 ^b^	0.58 ± 0.01 ^b^	35.75 ± 0.60 ^c^	77.62 ± 0.05 ^c^	91.22 ± 0.01 ^c^	91.17 ± 0.01 ^c^	1.30 ± 0.01 ^c^
SPS + 6% CNC	0.519 ± 0.003 ^b^	3.08 ± 0.12 ^d^	70.64 ± 0.43 ^c^	0.67 ± 0.05 ^ab^	31.50 ± 0.18 ^d^	75.08 ± 0.01 ^d^	91.35 ± 0.04 ^b^	91.31 ± 0.04 ^b^	1.44 ± 0.04 ^b^
SPS + 8% CNC	0.526 ± 0.002 ^a^	2.15 ± 0.01 ^e^	68.83 ± 0.18 ^d^	0.76 ± 0.05 ^a^	29.51 ± 0.18 ^e^	67.97 ± 0.03 ^e^	92.23 ± 0.04 ^a^	92.19 ± 0.05 ^a^	2.34 ± 0.09 ^a^

Values are expressed as the average of triplicate tests ± standard deviation. Different superscript letters in each column indicate significant differences at *p* ≤ 0.05. WVP is an abbreviation for water vapor permeability.

## Data Availability

The original contributions presented in this study are included in the article. Further inquiries can be directed to the corresponding author.
